# Organoid technology and applications in lung diseases: Models, mechanism research and therapy opportunities

**DOI:** 10.3389/fbioe.2022.1066869

**Published:** 2022-12-08

**Authors:** Jingyao Chen, Feifei Na

**Affiliations:** State Key Laboratory of Biotherapy and Cancer Center, Department of Thoracic Oncology, West China Hospital, Sichuan University, Chengdu, China

**Keywords:** lung diseases, organoids, SARS-CoV-2, lung cancer, drug discovery

## Abstract

The prevalency of lung disease has increased worldwide, especially in the aging population. It is essential to develop novel disease models, that are superior to traditional models. Organoids are three-dimensional (3D) *in vitro* structures that produce from self-organizing and differentiating stem cells, including pluripotent stem cells (PSCs) or adult stem cells (ASCs). They can recapitulate the *in vivo* cellular heterogeneity, genetic characteristics, structure, and functionality of original tissues. Drug responses of patient-derived organoids (PDOs) are consistent with that of patients, and show correlations with genetic alterations. Thus, organoids have proven to be valuable in studying the biology of disease, testing preclinical drugs and developing novel therapies. In recent years, organoids have been successfully applied in studies of a variety of lung diseases, such as lung cancer, influenza, cystic fibrosis, idiopathic pulmonary fibrosis, and the recent severe acute respiratory syndrome coronavirus-2 (SARS-CoV-2) pandemic. In this review, we provide an update on the generation of organoid models for these diseases and their applications in basic and translational research, highlighting these signs of progress in pathogenesis study, drug screening, personalized medicine and immunotherapy. We also discuss the current limitations and future perspectives in organoid models of lung diseases.

## 1 Introduction

Owing to natural lung aging and the constant exposure of humans to air pollution, cigarettes, and viruses, lung diseases are the leading cause of morbidity and mortality worldwide (Calvert and Ryan Firth, 2020; Lozano et al., 2012). Lung diseases range from common diseases such as lung cancer, influenza, and asthma to genetic disease cystic fibrosis (CF) and the novel coronavirus disease COVID-19 (Gruzieva et al., 2022). Very few treatment options are available for patients with advanced and recurrent diseases. Thus, lung diseases pose a serious threat to human health globally. Developing *in vitro* and *in vivo* model systems that accurately reflect the genetic and phenotypic specificity of a disease is essential for studying genetic and molecular alterations and developing novel treatments (Bleijs et al., 2019).

Traditional lung disease models, including two-dimensional (2D) cell lines, genetically engineered mouse models (GEMM), and patient-derived xenografts (PDXs), have contributed to revealing pathogenesis and evaluating treatment (Kim et al., 2020a). However, 2D culture substrates cannot recapitulate the variety of cell-cell, cell-matrix interactions experienced by cells within the human organand provide the complex extracellular environment necessary to model the organ *in vivo* (Sundarakrishnan et al., 2018a). The extensive application of GEMM is restricted for several reasons, including difficult technique and complex breeding processes (Cheon and Orsulic, 2011). The PDX model cannot faithfully recapitulate the entire process of tumorigenesis, from normal cells into fully transformed malignancies, which limits its application (Byrne et al., 2017). Therefore, more advanced platforms are required. Recently developed organoid technologies have led to the development of novel and more valid models for the study of human disease (Wang et al., 2017; Weeber et al., 2017; Drost and Clevers, 2018; Di Lullo and Kriegstein, 2017; Kraiczy and Zilbauer, 2019; Clevers, 2016). Organoids are three-dimensional (3D) *in vitro* culture models derived from self-organizing stem cells and can recapitulate the *in vivo* cellular components, functionality, and genetic characteristics of the original tissues (Dutta et al., 2017). Compared with 2D cultures, organoids provide more fundamental insights into pathogenesis and offer new approaches for the diagnosis and treatment of diseases (Ballard et al., 2019; Drost and Clevers, 2017; Simian and Bissell, 2017). Lung organoids can be established from healthy tissue and patient-derived disease tissue. In establishing a patient-derived lung cancer model, the growth rate of 2D lung cancer cells notably slowed down after passage five, which limited availability in basic and translational research. Lung cancer organoid models have a greater long-term potential and maintain tissue architecture during expansion *in vitro* (Kim et al., 2019). 2D cultures of lung fibroblasts derived from idiopathic pulmonary fibrosis (IPF) patients failed to demonstrate the features seen in patients (Wilkinson et al., 2017a; Pierce et al., 2007). 2D cell cultures only partially exhibit the morpho-molecular pattern required for viral tropism (Rosellini et al., 2019). Lung organoids represent an important link between traditional 2D lung cell cultures and lung tissues, as they are more physiologically and mechanically similar to tissues than monolayer culture models (Fatehullah et al., 2016). Moreover, combined with genome editing technology, lung organoid culture suggests a new strategy for generating primary and orthotopic lung cancer models, which is very convenient and time-saving compared to GEMMs.

Lung organoids provide a novel tool for studying the development of lung diseases and new therapies (van der Vaart and Clevers, 2021; Lancaster and Knoblich, 2014). In this review, we discuss recent advances in lung organoid technology for lung diseases, including lung cancer, infectious disease, IPF, and CF. We further reviewed the applications of organoid technology in drug development and personalized medicine. We critically appraised the value of lung organoids as disease models (Table1). We also discuss the limitations of this study and consider possible solutions.

**TABLE 1:** Key studies of lung disease model using organoids.

Disease modelled	Disease pheotype	Starting cell population	Cell type composition or markers detected	Onset of organoid formation	Expansion	Success rate	Mice xenotransplanation	Reference
NSCLC	LUAD; LUSC	ASC	TTF-1, TP63, CK5/6	Digestion	Short-term (1–3 months, 1–9 passages)	88% (57/65)	+	PMID: 31694835
					Long-term (>3 months, >10 passages)			
NSCLC and SCLC	Adenocarcinoma, Squamous cell carcinoma, Adenosquamous carcinoma, Large cell carcinoma, Small cell carcinoma	ASC	Adenocarcinoma: NAPSIN, TTF-1, CK7;Squamous cell carcinoma: p63, CK5/6; Adenosquamous carcinoma: CK7, CK5/6, p63; Small cell carcinoma: CD56, TTF-1, SYP	Digestion	Over 6 months	87% (lung cancer samples)	+	PMID: 31488816
						70% (cryopreserved organoids)		
SCLC	SCLC	ASC	CD56, SYP, CHR	Digestion	Over 9 months	80% (8/10)	−	PMID: 33572899
SCLC	SCLC	ASC	ASCL1, SYP, CHGA, TTF1	Gene editing		NA	+	NC
Parainfluenza	HPIV3	human PSC	AT2 cell marker SPC, EpCAM, Viral component NP	Infection	At least 32 days	NA	−	PMID: 31064833
Influenza	IBV	ASC	Acetyl-α-tubulin, SCGB1A1/CC10, MUC5AC, p63-α, IBV component	Infection	NA	NA	−	PMID: 31097520
Influenza	IAV	ASC	ACCTUB, FOXJ1, MUC5AC, p63, CC10, Viral component NP	Infection	NA	NA	−	PMID: 29891677
Influenza	IAV (H1N1, H7N9, H5N6, H5N1)	ASC	p63a, Acetyl-α-tubulin, MUC5AC, SCGB1A1/CC10, Influenza NP	Infection	NA	NA	NA	PMID: 30001996
Covid-19	SARS-CoV-2 (BetaCov/Korea/KCDC03/2020)	ASC	pro-SFTPC, HTII-280, ABCA3, CRB3, SCRIB, AQP5, AEGR, TP63, ACE2, MX1, TMPRSS2, Viral components (NP or dsRNA)	Infection	Over 10 months (expansion of human AT2 cells)	NA	NA	PMID: 33142113
Covid-19	SARS-CoV-2 USA-WA1/2020	human AT2 cells/pneumocytes derived from primary lung tissue	NKX2-1, SFTPC, SFTPB, HTII-280, DC-LAMP, EPCAM, Polarity marker ZO1	Infection	Over 10 months (expansion of human AT2 cells)	NA	NA	PMID: 33128895
Covid-19	SARS-CoV-2 isolate USA-WA1/2020	iPSC	ECAD, VIM, SMA, ACTUB, p63, FOXJ1, SP-B, TMPRSS2, SP-C, ACE2	Infection	60 days	NA	NA	PMID: 33631122
Covid-19	SARS-CoV-2, isolate USA-WA1/2020 (NR-52281)	human PSC	SP-B, SP-C, ACE2, SARS-S	Infection	NA	NA	+	PMID: 33116299
Covid-19	SARS-CoV-2 (isolate BetaCoV/Munich/BavPat1/2020)	Progenitor cells derived from human fetal lungs	AcTUB, Nucleoprotein, CC10, MUC5AC, HTII-280, LPCAT1, SFTPC, HOPX, HTI-56, TP63, ACE2, TMPRSS2	Infection	NA	NA	NA	PMID: 33283287
Covid-19	SARS-CoV-2 (isolate BetaCoV/Munich/BavPat1/2020)	ASC	NP, ZO-1, AcTub	Infection	NA	NA	NA	PMID: 33393462 and 33835028
Covid-19	SARS-CoV-2 (USA-WA1/2020)	AT2 or KRT5+ basal cells	KRT5, SFTPC, SCGB1A1, SFTPC, HTII-280, SFTPC, HTI-56, ACE2, AcTUB, TNFRSF12A	Infection	6 months	NA	NA	PMID: 33238290
Covid-19	SARS-CoV-2 strain USA-WA1/2020	ASC	SFTPB, SFTPC, KRT5, TTF1, AcTub, MUC5AC, CC10, ACE2, AQP5, Na/K ATPase	Infection	NA	NA	NA	PMID: 34463615
IPF	IPF	human PSC	PDGFRA, ECAD, EPCAM, KRT8, NKX2.1, FOXA1, p63, FOXA2, SOX9, SOX2, MUC1, MUC5AC, SFTPC, SFTPB, ABCA3, VIM, CD90, COL4, Fibronectin, PDGFRA, PDGFRB, Vimentin, Collagen I, Collagen III, SLUG, SMA	Gene editing	170 days	NA	+ (WT LBOs)	PMID: 28436965 and 31216486
IPF	IPF	human PSC	IL6, CXCL8, SERPINE1, SFN, GDF15, MMP7	Bleomycin treatment	NA	NA	NA	PMID: 34798066
IPF	IPF	iPSC	Vimentin, Collagen I, ɑ-SMA	TGF-β treatment	NA	NA	NA	PMID: 28191779
CF	CF	ASC	KRT5, Acetyl-α-tubulin, MUC5AC, SCGB1A1, (periodic acid-chiff) PAS, β-tubulin, F-actin	Digestion	NA	62%	NA	PMID: 30643021
Cryptosporidiosis	Cryptosporidiosis	ASC	Lung organoid consists of basal cells, ciliated cells, goblet cells and club cells. gp-15, Crypt-a-Glo	Microinjection	NA	NA	NA	PMID: 29946163 and 31566619

NA, not available; SCLC, small cell lung cancer; NSCLC, non-small cell lung cancer; LUAD, lung adenocarcinoma; LUSC, lung squamous cell carcinoma; IPF, idiopathic pulmonary fibrosis; CF, cystic fibrosis; HPIV3, human parainfluenza virus type 3; IAV, influenza A virus; IBV, influenza B virus; ASC, adult stem cell; PSC, pluripotent stem cell; iPSC, induced pluripotent stem cell; TGF-β, transforming growth factor-β.

## 2 A brief history of lung organoids

**FIGURE 1:**
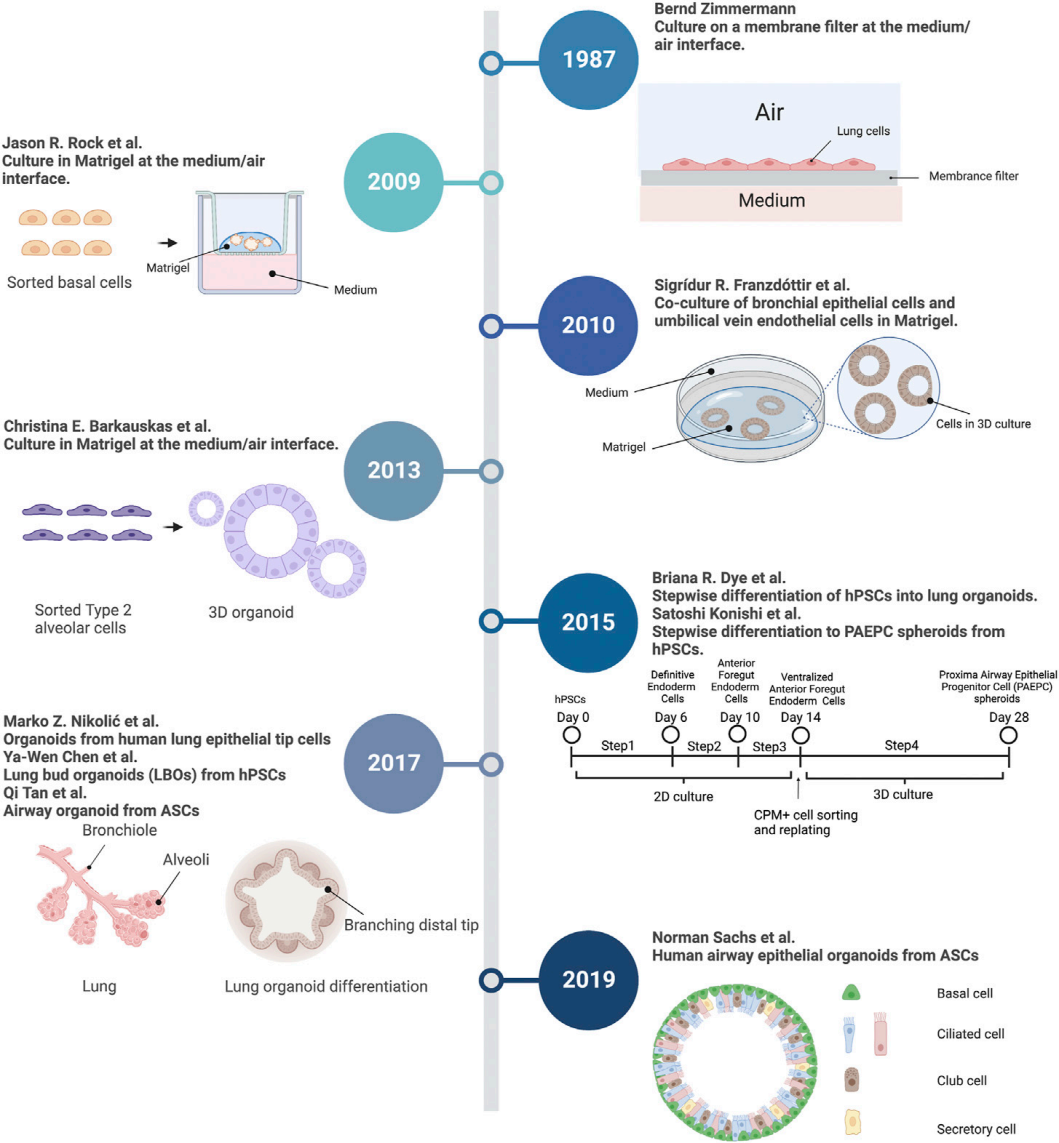
Timeline of techniques and experiments in lung organoid field. A summary of key events in the history contributing to lung organoid methodology. 3D, 3-dimensional; hPSCs, human pluripotent stem cells; ASCs, Adult stem cells; PAEPC, proximal airway epithelial progenitor cells. Created with BioRender.com.

Lung organoid methods were build upon the extensive foundation of classic developmental biology and cell experiments (Figure 1). In 1987, Zimmermann (1987) described a lung organoid culture system. When cultured on the medium/air interface at a high density, the mouse fetal lung cells differentiated and formed organoid structures: an alveolar-like lumen and a basal lamina. Developmental biology has revealed that basal cells emerge in the early stages of human lung development and serve as an important stem cell lineage (Hajj et al., 2007). In 2009, researchers described an *in vitro* 3D organoid culture system (Rock et al., 2009). Unlike the previous air-liquid interface (ALI) culture, the 3D system used a supportive extracellular matrix (Matrigel), in which basal cells self-renew and differentiate into “clonal sphere” (organoid). The spheres consisted of KRT14 + basal and KRT8+ luminal cells. Similarly, Franzdottir et al. (2010) developed a human bronchial epithelial cell line that maintained basal cell phenotype. The co-culture of this cell line and endothelial cells generated branching bronchioalveolar-like structures in 3D Matrigel culture. Barkauskas et al. (2013) revealed that alveolar type 2 epithelial (AT2) cells can also self-renew and differentiate into “alveolospheres” which contain both AT2 and alveolar type 1 epithelial (AT1) cells. Early lung organoid studies showed that progenitors in human lung could differentiate and organize into multicellular spheres. More recently, several studies successfully established lung organoids derived from adult stem cells (ASCs) and pluripotent stem cells (PSCs) (Dye et al., 2015; Konishi et al., 2016; Nikolic et al., 2017; Chen et al., 2017; Tan et al., 2017; Sachs et al., 2019). PSCs include embryonic stem cells (ESCs) and induced pluripotent stem cells (iPSCs) (Dye et al., 2015). These lung organoids are capable of self-renewal and self-organization and are composed of multiple cell types. Using a stepwise differentiation strategy, proximal airway epithelial progenitor cell spheroids were generated from human PSCs (Konishi et al., 2016). Similarly, human PSCs differentiate into lung organoids consisting of basal cells and immature ciliated cells surrounded by smooth muscle and myofibroblasts, as well as an alveolar-like domain with appropriate cell types (Dye et al., 2015). In addition to human lung organoids, human pluripotent stem cells (hPSCs) also could be differentiated into bud tip progenitor organoids to explore epithelial fate decisions (Miller et al., 2019). Also, ASC-derived human lung organoids contain basal, secretory, and multi‐ciliated cells (Sachs et al., 2019). After transplantation into injured mouse lungs, normal lung organoids exhibited progenitor cell functions, suggesting that they might be applied in regenerative medicine (Lee et al., 2014), (Louie et al., 2022). Finally, these systems were subsequently adapted to generate human and mouse normal lung organoids, and organoids derived from patients with lung disease.

## 3 Organoids modeling lung cancer

Studies have found that almost a quarter of all cancer-related deaths are caused by lung cancer (Islami et al., 2018). The risk of lung cancer is particularly prominent in men aged ≥40 years and women aged ≥60 years. Lung cancer causes far more deaths than breast cancer, prostate cancer, and colorectal cancer (CRC) combined (Siegel et al., 2021). Therefore, it is important to study the processes of tumorigenesis, tumor maintenance, and therapeutic sensitivity. Organoid technologies provide a powerful platform for investigating mechanistic processes and developing novel treatment strategies (Wang et al., 2020). Lung cancer is a complex disease composed of different molecular and histological types, including non-small cell lung cancer (NSCLC) and small cell lung cancer (SCLC) (Hirsch et al., 2017). In the following sections, we discuss advances in organoid technologies for modeling NSCLC and SCLC.

### 3.1 Organoids modeling NSCLC

NSCLC accounts for approximately 85% of lung cancers (Herbst et al., 2018). For NSCLC patients, the use of small-molecule inhibitors and immunotherapy has led to increased survival benefits (Camidge et al., 2019). However, the overall survival rate of NSCLC remains low (Herbst et al., 2018). Several groups have identified cell culture conditions that favor the expansion of NSCLC organoids derived from patient tissues and PDXs (Kim et al., 2019; Shi et al., 2020; Takahashi et al., 2019). Shi et al. (2020) described a culturing protocol that enables the generation of short-term (1–3 months, 1–9 passages) and long-term (>3 months, >10 passages) NSCLC organoids. The organoids were maintained in culture for short-term and long-term success rates of 72% (47/65) and 15% (10/65), respectively. Both short-term and long-term established NSCLC organoids recapitulated the histological features of the patient and the PDX tumor. Whole exome and RNA sequencing revealed that NSCLC organoids also maintained genomic heterogeneity in parental lung cancer. Organoids respond to drugs based on genomic alterations. Similar to the results of previous studies, NSCLC organoids with KRAS mutation and amplification were much more sensitive to the MEK inhibitors trametinib and selumetinib than organoids with wild-type KRAS. Thus, organoids can be used to predict patient-specific drug responses *in vitro*, based on genetic alterations. Kim and colleagues banked 80 lung cancer organoid lines, of which more than half were NSCLC subtype (Kim et al., 2019). NSCLC organoids displayed typical histological features seen in the original patient tumor tissue, such as large glandular patterns in adenocarcinoma-derived organoids. They also recapitulated the diagnostic markers of the original cancer tissues and maintained the genomic alterations in the original tumors. Intriguingly, the programmed death-ligand 1 (PD-L1) expression patterns of tumor organoids were the same as those of the original tumor tissues, suggesting that organoids co-cultured with immune cells may be used to study tumor immune interactions.

### 3.2 Organoids modeling SCLC

SCLC, a highly lethal lung cancer subtype with a 5-year survival rate of <10% (Bunn et al., 2016), accounts for 10%–15% of all lung cancers (Basumallik and Agarwal, 2021). Kim et al. (2019) established lung cancer organoids from patient tissues as *in vitro* models representing individual patients. They banked 80 lung cancer organoid lines, including five lines from SCLC. These SCLC organoids recapitulated the tissue architecture of densely packed small round tumor cells with scanty cytoplasm and granular nuclei and maintained the expression of the SCLC diagnostic markers CD56, synaptophysin, and TTF-1. Choi et al. generated SCLC patient-derived tumor organoids. These tumor organoids displayed the typical architecture of the original tumor tissues. Furthermore, SCLC organoids were subjected to long-term expansion. The addition of WNT3A or R-spondin1 to the culture medium promoted the long-term survival of SCLC organoids. These results indicate that activation of the Wnt/β-catenin pathway is essential for the long-term expansion of SCLC organoids. After long-term expansion, SCLC organoids maintained similar molecular characteristics, genetic profiles, and morphological architectures as the original tumor tissues (Choi et al., 2021a). Using these organoids, drug screening for therapeutic and toxic effects may contribute to the development of novel therapeutic strategies.

Combined with CRISPR/Cas9 technology, lung organoids can be used to model SCLC tumorigenesis and metastasis *in vivo*. Na et al. (2022) developed a strategy to generate primary, orthotopic, and driver-defined SCLC in mice with genome-edited lung organoids. Similar approaches have been used to model breast cancer, colorectal cancer, and others (Nanki et al., 2018; Duarte et al., 2018; Dekkers et al., 2020; Matano et al., 2015). Superior to the cell line xenograft model and PDX model, these novel models recapitulate the molecular and pathohistological characteristics of human diseases, especially the entire process of multiple-step tumorigenesis from normal cells into malignancies. This SCLC model is driven by *Trp53* and *Rb1* deficiencies, together with *Myc* overexpression. In this SCLC model, the tumor cells were positive for multiple SCLC diagnostic markers such as TTF1, ASCL1, SYP, CHGA, and KI67. Notably, massive distal metastases, the key feature of human SCLC, are observed in multiple organs of this model. Additionally, this strategy can introduce any driver mutation to identify their biological functions in the initiation and progression of SCLC, which is more convenient than the traditional methods.

## 4 Organoids modeling infectious lung disease

The growth and culture of pathogens are now possible through the establishment of organoids (Azar et al., 2021). Organoids can serve as models for studying pathogenesis, immune responses, respiratory host-pathogen interactions, and drug screening. Intestinal organoids have been widely applied in studies of human noroviruses (Estes et al., 2019; Ettayebi et al., 2016; Haga et al., 2020) and Zika virus (Qian et al., 2017; Watanabe et al., 2017). Airway organoids have also been used to model various infectious lung diseases caused by pathogens (Chen et al., 2017), including severe acute respiratory syndrome coronavirus-2 (SARS-CoV-2), influenza virus, respiratory syncytial virus (Rijsbergen et al., 2021a; Rijsbergen et al., 2021b), parainfluenza virus (Porotto et al., 2019; Rijsbergen et al., 2022), Mycobacteria (Iakobachvili et al., 2022), *Streptococcus* (Sempere et al., 2022), and the protozoan parasite *Cryptosporidium*. Here, we focused on influenza, COVID-19, and cryptosporidiosis organoid models.

### 4.1 Organoids modeling influenza

Influenza is an acute viral respiratory infection that causes significant morbidity and mortality worldwide (Gaitonde et al., 2019). Three distinct types of influenza viruses infect humans: influenza A, B, and C. There is no robust *in vitro* model for predicting the infectivity of influenza viruses in humans, which limits the research on influenza. Based on the long-term expanding 3D human airway organoids, Zhou et al. (2018) established improved 2D monolayer culture conditions for the differentiated airway organoids. Both the 2D differentiated airway organoids and the previously reported 3D system can morphologically and functionally simulate the human airway epithelium and be infected by influenza A viruses. Notably, the highly human-infective influenza A virus (IAV) isolate replicated dramatically, with a higher viral titer than the mild isolate. Thus, airway organoids can be used to rapidly assess the infectivity of emerging respiratory viruses in humans. The Michael group has used IAVs and influenza B viruses (IBVs) to infect human airway organoids derived from ASCs. They found that replication efficiencies in airway organoids were similar to those in human bronchial explants. Low tissue availability limits the applicability of the *ex vivo* explant model. However, airway organoids can self-renew and maintain tissue architecture during long-term expansion *in vitro*. Histology has revealed that airway organoids mimic the composition, cellular diversity, and organization in human airways (Bui et al., 2019; Hui et al., 2018). The successful establishment of these platforms provides a foundation for predicting the infectivity of influenza viruses and studying the interactions between the host and pathogen.

### 4.2 Organoids modeling COVID-19

With the recent spread of the COVID-19 pandemic, respiratory models have been rapidly established to study the biology and pathogenesis of SARS-CoV-2 infection (Asselah et al., 2021). Organoid models have been used not only in 3D structures but also in 2D ALI culture systems. Salahudeen et al. (2020) established a long-term organoid culture of the distal human lung, including AT2 and basal stem cells, and used this system to validate functional progenitor cells and to model SARS-CoV-2 infection, (Mallapaty, 2021). Similarly, Youk et al. (2020) developed a feeder-free, long-term, 3D culture technique for human AT2 cells derived from primary human lung tissues. This system appears to be more permissive to SARS-CoV-2 infection than the systems previously reported. Tindle et al. (2021) presented an adult lung organoid model that was complete with proximal airway and distal alveolar cell types, which were critical for viral infection and host immune response. Lamers et al. (2021a) reported that 2D airway organoid-derived ALI differentiated cultures allow efficient SARS-CoV-2 replication. This model reveals that SARS-CoV-2 entry into human airway organoids is serine protease-mediated and facilitated by the multibasic cleavage site (MBCS) (Mykytyn et al., 2021). Furthermore, they investigated whether mutations in SARS-CoV-2 spike MBCS could be prevented in this model. The results revealed that the 2D ALI airway organoids prevent SARS-CoV-2 multibasic cleavage site cell culture adaptation; thus, this model can be used for SARS-CoV-2 propagation if new variants emerge that are not genetically stable in cell lines (Lamers et al., 2021b). Airway organoids derived from hPSCs can also be used to model SARS-CoV-2 infections. Tiwari et al. generated human iPSC-derived lung organoids containing epithelial cells, AT1 and AT2 cells. These organoids express host cell receptor angiotensin-converting enzyme 2 (ACE2) and transmembrane serine protease 2 (TMPRSS2). They are also permissive to SARS-CoV-2 infection (Tiwari et al., 2021). In 2D airway organoids, the Omicron variant showed more robust replication than the WT virus, as seen in the respiratory tract (Chiu et al., 2022).

### 4.3 Organoids modeling cryptosporidiosis

For protozoan parasites, non-existent or inappropriate animal models limit the study of their biology and interactions with the host (Klotz et al., 2012). *Cryptosporidium* causes a diarrheal disease called cryptosporidiosis, which is a major cause of diarrhea in children (Bouzid et al., 2013; Thompson et al., 2005). Traditional 2D culture systems of cell lines only allow short-term infection (<5 days) and support an incomplete life cycle of the parasite (Dutta et al., 2019; Muller and Hemphill, 2013; Karanis and Aldeyarbi, 2011; Karanis, 2018). Adult-stem-cell-derived organoids replicate the physiology of their original tissues, providing possible conditions for infection and replication of protozoan parasites (Clevers, 2016). Heo et al. (2018) revealed that *Cryptosporidium* could infect intestinal and lung organoids derived from healthy human tissues. They introduced *Cryptosporidium* oocysts into organoids by microinjection. Compared to other methods, microinjection can maintain the 3D structure of organoids and allow precise control of parasite volume and direct apical-side contact of microbes (Dutta et al., 2019). About 100–1,000 oocysts were injected into each organoid. *Cryptosporidium* can complete its full life cycle by generating new oocysts within the small intestinal and lung organoids. The new oocysts were infectious in the mouse experiments. This system supports the induction of type 1 interferon signaling, which is considered an antiparasitic effector (Barakat et al., 2009). This study established a physiologically relevant *in vitro* model system with organoids that are adaptable for studying parasitic biology and developing antiparasitic drugs.

## 5 Organoids modeling pulmonary fibrosis

In addition to cancer and infectious diseases, lung organoids from human PSCs or ASCs can model certain aspects of human pulmonary fibrosis, including IPF and CF (Danahay et al., 2015; Kobayashi et al., 2020; Strunz et al., 2020; Jacob et al., 2017; McCauley et al., 2017). Pulmonary fibrosis results from toxic, infectious or drug-induced injuries. Respiratory failure is the most common cause of pulmonary fibrosis-related death. Several genetic variants have been found to be associated with the progress of pulmonary fibrosis (Thannickal et al., 2004). Despite some breakthroughs in research, the clinical treatment strategy of pulmonary fibrosis remains very limited. Organoid technology contributes to mechanism research and drug development.

### 5.1 Organoids modeling idiopathic pulmonary fibrosis

IPF is a particularly severe form of pulmonary fibrosis, with a median survival of 3–5 years after diagnosis. There are only two approved treatments for IPF (pirfenidone and nintedanib), which merely decelerate lung function decline (Sundarakrishnan et al., 2018b). *In vitro* models have contributed to the discovery of antifibrotic drugs and the study of pathogenesis. Surolia et al. (2017) cultured 3D organoids (pulmospheres) derived from IPF patients. Pulmospheres are composed of cells derived from patient lung biopsies, demonstrating the presence of α-SMA–positive, SPC-positive, CD31-positive, and CD11b-positive cells. Immunofluorescence staining demonstrated that the cells in the IPF pulmospheres were supported and surrounded by extracellular matrix proteins, which recapitulated the *in vivo* architectural features. Additionally, the IPF pulmospheres in 3D culture have similar expression levels of cellular differentiation marker genes with those *in vivo*. Whereas, the same cells grown in 2D culture have less similarities (Surolia et al., 2019).

Genetic modification or stimulation of growth factors can also confer the pathological features of IPF in normal organoids. In lung bud organoids, the mutation of *HPS1*, *HPS2*, or *HPS4* leads to fibrotic changes, showing accumulation of extracellular matrix and mesenchymal cells (Chen et al., 2017). Deletion of IL-11 prevented fibrosis in *HPS4* ^−/−^ organoids, indicating IL-11 as a potential therapeutic target (Strikoudis et al., 2019). Transforming growth factor-β (TGF-β) induces fibroblasts to obtain a myofibroblast phenotype (Wynn and Ramalingam, 2012). Wilkinson et al. (2017b) generated a model of progressive scarring that resembled human IPF by treating fetal lung fibroblast organoids or inducing pluripotent stem cell-derived mesenchymal cell organoids with TGF-β. In another example, bleomycin treatment induced IPF phenotypes such as fibroblast activation, cellular senescence and morphological change in human PSC-derived alveolar organoids (Suezawa et al., 2021).

### 5.2 Organoids modeling cystic fibrosis

CF is a multisystemic autosomal recessive disease with an incidence of 1/3,000–10,000 newborns, which is caused by a wide variety of mutations in the CF transmembrane conductance regulator (CFTR) gene (Calucho et al., 2021). McCauley et al. (2017) generated CF patient-specific iPSC-derived airway organoids. They revealed the rapidity and stage specificity of Wnt-driven proximodistal airway patterning and generated the “low-Wnt” protocol to derive organoids from purified NKX2-1+ progenitors. A functional assessment revealed that iPSC-derived organoids exhibited CFTR-dependent swelling in response to forskolin. ASC-derived airway organoids of CF patients were established by Sachs et al. (2019). Airway organoids from ASCs recapitulate central disease features and swell upon modulation of CFTR as well as activation of TMEM16A. The pathological manifestations of CF occur in multiple organs, such as the lungs, sinuses, pancreas, intestines, hepatobiliary tree, and vas deferens (Scott, 2013). Thus, rectal, intestinal, and naso-organoids are used as *in vitro* models for CF (Calucho et al., 2021; Berkers et al., 2019). These CF organoid models will facilitate gene functional studies, drug development, and personalized medical approaches for CF.

## 6 Organoids modeling goblet cell metaplasia

GCM, an increase in the number of goblet cells and an overproduction of mucus, is a key feature common to many airway diseases, such as asthma, chronic obstructive pulmonary disease (COPD), and CF (Danahay et al., 2015; Fahy and Dickey, 2010). In 3D cultures, airway basal cells differentiate into goblet and ciliated cells and form “bronchospheres.” IL-13 induces a mucus hypersecretory phenotype in bronchospheres. Henry et al. developed a screenable 3D culture system of airway epithelial morphogenesis using bronchospheres. Through high-throughput screening, they found that similar to IL-13, many inflammatory cytokines could bias airway basal cell fate to drive GCM, a phenotype similar to GCM seen in many airway diseases. Notch2 has been identified as a key node required for GCM. Therefore, Notch2 may be a novel therapeutic target to prevent the development of GCM in respiratory diseases.

Li et al. (2021a) found that *LKB1* expression was downregulated in the lungs of patients with COPD. An organoid culture study has demonstrated that *Lkb1* deficiency drives airway goblet cell differentiation and pulmonary macrophage recruitment. The *Retnla* expression levels were increased in club and goblet cells in the absence of LKB1. RELM-α, the protein encoded by *Retnla*, promotes the expression of goblet cell markers Muc5Ac, ClCa3, and Foxa3 in 3D organoid cultures. These results reveale that LKB1 deficiency-mediated RELM-α upregulation promotes airway GCM, suggesting that restoring LKB1 expression is a potential therapeutic strategy to treat respiratory disorders associated with GCM.

## 7 Applications of organoid technology in lung diseases

**FIGURE 2:**
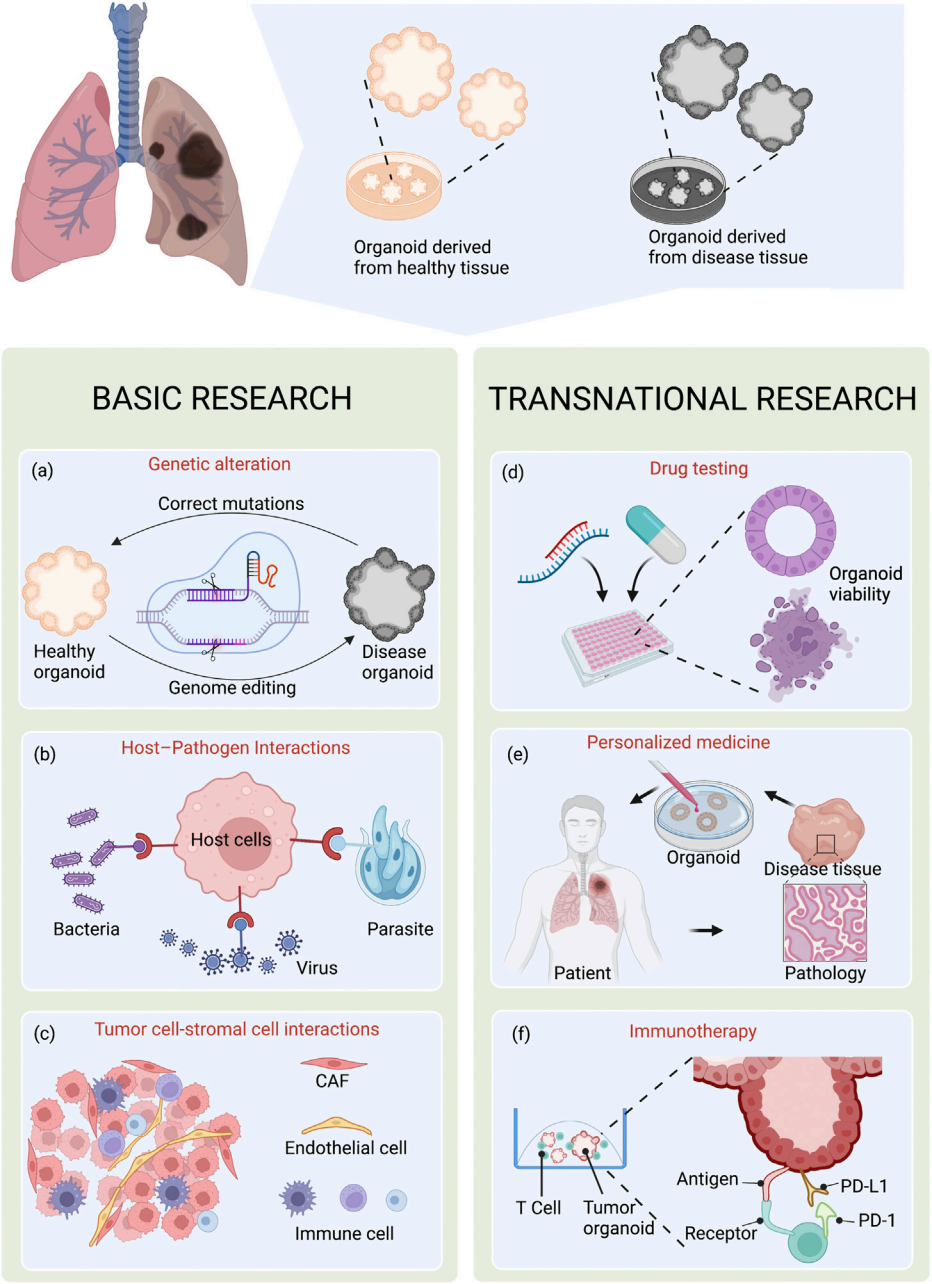
Applications of organoid technology in lung diseases. Lung organoids can be established from healthy tissue and patient-derived disease tissue. 3D organoid cultures have great value in basic and translational research. **(A)**Combined with gene editing technique CRISPR/Cas9, organoid system provides ideal models for investigating genetic diseases caused by genetic alteration. **(B,C)** Organoid technology provides *in vitro* models for studying host-pathogen interactions and tumor cell-stromal cell interactions. **(D,E)** At the preclinical stage, organoids can also be used in drug development, including high-throughput drug screening and personalized drug. **(F)** In addition, 3D co-culture of tumor cells and T cells have the potential to study tumor immumotherapy. Created with BioRender.com.

The *in vitro* lung organoid model is an excellent system for a wide range of basic and translational research (Figure 2).

### 7.1 Lung organoids for basic research

Organoids recapitulate the features of the primary cell population and mimic the responses to genetic alterations and environmental effects. The organoid system allows researchers to intensively study the alterations at the genetic and molecular levels, as well as in the microenvironment during lung disease development.

#### 7.1.1 Genetic alterations

Organoids can be used to model and study the initiation and progression of lung diseases. Combined with CRISPR/Cas9 gene editing technology, lung organoids are well suited to identify genes that play important roles in disease development. As a striking example, Na et al. (2022) generated an organoid-derived SCLC model and found that *Kmt2c* deficiency promoted SCLC tumorigenesis and metastasis. This study was the first to reveal the function of *KMT2C* gene in SCLC. Differently from previously reported in leukemia (Chen et al., 2014), *KMT2C* deficiency promotes SCLC tumorigenesis and metastasis through DNMT3A-mediated epigenetic reprogramming. Strikoudis et al. (2019) found that the deletion of *HPS1*, *HPS2*, or *HPS4* in lung organoids generated from ESCs promoted fibrotic changes in lung organoids. However, the deletion of *HPS8* did not. Zheng et al. (2022) identified *DNAH9* mutation in primary cilia dyskinesia patient, and constructed *Dnah9* knock-down organoids, which displayed typical Primary cilia dyskinesia phenotypes: reduced ciliary beating and increased secretion of inflammatory factors.

The mutations of the *CFTR* gene cause CF (Stutts et al., 1995). CF organoid models could contribute to *CFTR* gene functional studies. Organoids derived from patients with CF allow the assessment of *CFTR* function in an organoid swelling assay. Dekkers et al. (2013) found that the function of the *CFTR* F508del mutant protein was restored by *CFTR*-restoring compounds. Wong et al. (2022) revealed the conductance defect of the rare *CFTR* mutation R352Q. Organoids derived from CF patients are also used to generate biobank for the study of gene correction by adenine base editors and *CFTR* repair (Geurts et al., 2020). *CFTR* is an attractive target for gene editing approaches. Gene therapy has been used to correct the *CFTR* locus using CRISPR/Cas9 and homologous recombination in CF patient-derived organoids. In these studies, organoids are used as a tool to model lung cancer and fibrotic lung disease and identify disease-associated genes.

#### 7.1.2 Pathogenic infection

Because organoids represent most cellular components found in the organ of origin, they are widely applied to study the interactions between host and pathogen, particularly tropism and host immune response. Lung organoids derived from human PSCs have been used to study the cell tropism of SARS-CoV-2. Pei et al. (2021) demonstrated that SARS-CoV-2 infects ciliated, club, and AT2 cells. ASGR1 and KREMEN1 were identified as alternative receptors of SARS-CoV-2 and played essential roles in ACE2-independent virus entry (Gu et al., 2022). In SARS-CoV-2-infected airway organoids, HIF1ɑ signaling regulated glycolysis, and GW6471 blocked infection by inhibiting the HIF1ɑ-glycolysis axis (Duan et al., 2021). Using human ASC-derived lung organoids, Bui et al. (2019) reported that IBVs infected various cell types, including ciliated, club, goblet, and basal cells. Similarly, human airway organoids have been employed in studying the cell tropism of IAV (Hui et al., 2018). H1N1, H7N9, and H5N6 viruses infected ciliated cells and goblet cells, but not basal cells in organoids, as observed in bronchus explants.

On the other hand, these models that mimic the cellular response to pathogen infection are crucial for studying the pathogenesis of the pathogen and developing vaccines and therapeutics. Katsura et al. (2020) reported an alveolosphere culture system for the propagation and differentiation of human AT2 cells. This system also supports the immune response to SARS-CoV-2 infection, and transcriptome analysis of infected organoids mirrors inflammatory responses in COVID-19 lungs. Low-dose interferon pre-treatment has proved to block SARS-CoV-2 replication in organoids, suggesting an effective therapy for COVID-19. Similarly, Youk et al. (2020) developed a SARS-CoV-2 infection model in which the expression of interferon-associated genes (such as *IFI27* and *IFI6*) and proinflammatory genes (such as *DDX58* and *IL6*) significantly increased, indicating an innate immune response in organoids. In iPSC-derived lung organoids, interferons, cytokines, and chemokines are induced, and key genes of the inflammasome pathway are activated after infection (Tiwari et al., 2021). Salgueiro et al. (2022) found that cancer-derived lung organoids showed a more decreased innate immune response to influenza A virus than those derived from healthy tissue. Overall, these models offer a robust system for studying pathogen infection and developing effective therapies (Rodrigues Toste de Carvalho et al., 2021).

#### 7.1.3 Tumor cell-stroma cell interactions

It is becoming well established that interactions between the stroma and tumor cells play a critical role in tumor growth and progression (Bussard et al., 2016). As described by Lambrechts, 52 stromal subtypes have been identified in human lung tumors, including tumor-associated fibroblasts, endothelial cells, and tumor-infiltrating immune cells (Lambrechts et al., 2018). However, little is known about the mechanisms of interactions between the stroma and tumor cells. The organoid *in vitro* co-culture systems provide a novel method for studying these cell-cell interactions. Chan et al. developed an *in vitro* 3D NSCLC model comprising human lung adenocarcinoma cells, fibroblasts, endothelial cells, and NSCLC cancer stem cells. This model recapitulates the tumor microenvironment characteristics and tumor cell heterogeneity (Chan et al., 2016). Using the alginate microencapsulation strategy, Rebelo et al. (2018) established a 3D cell model enclosing three cellular components: NSCLC cells, cancer-associated fibroblasts, and monocytes. The “3D-3-culture” model recreated the activation of monocytes into tumor-associated macrophages *in vitro* and demonstrated the macrophage plasticity in response to chemotherapy and immunotherapy. Similarly, by co-culturing AT2 cells with macrophages in an organoid system, Lechner et al. (2017) revealed that type 2 innate lymphoid cells and lung macrophages supported alveolar epithelial stem cell proliferation and differentiation. Ferreira et al. (2018) engineered hybrid 3D multicellular tumor spheroids that combined A549, fibroblasts, and human mesenchymal stem cells from bone marrow (hBM-MSCs) with bioinstructive hyaluronan microparticles. In this *in vitro* model, hBM-MSCs exhibit dynamic interactions with cancer cells and fibroblasts, as observed *in vivo*. To study tumor metastasis, Ramamoorthy et al. (2019) developed a multicellular lung organoid model that resembles the architecture of metastatic tumors *in vivo*. This study provides an *in vitro* model for studying tumor metastasis and has significant utility in developing ideal therapeutic agents. These findings demonstrate the ability of organoids to recapitulate the interactions of tumor cell-stroma cell, suggesting the potential use of organoid models to develop novel therapies targeting the tumor microenvironment.

### 7.2 Lung organoids for clinical translational research

Organoids maintain the functions of primary tissues and recapitulate drug responses. Therefore, organoid technology may significantly improve therapeutic development. Below, we discuss the current applications of lung organoids in therapeutics and drug development and list the important reports about drug development based on lung organoids (Table 2).

**TABLE 2:** Therapy strategy development based on lung organoids.

Therapy	Drug	Target	Mechanism	Preclinical study or clinical trial	Reference
PARP inhibitor	Olaparib	BRCA2 p.W2619C NSCLC	NA	NA	PMID: 31488816
EGFR tyrosine kinase inhibitors	Erlotinib	EGFR-mutant NSCLC	NA	NA	PMID: 31488816
c-Met inhibitor	Crizotinib	EGFR-mutant/MET-amplified organoid NSCLC	NA	NA	PMID: 31488816
ERBB2 inhibitor and RET-fusion inhibitor	Poziotinib, pralsetinib	Lung adenocarcinoma harboring ERBB2 exon 20 insertions or RET fusions, respectively	NA	Clinical trial registration: NCT02609776	PMID: 34083237
EGFR-MET Bispecific Antibody	Amivantamab (JNJ-61186372)	EGFR Exon 20 Insertion NSCLC	Amivantamab Induces Antibody-Dependent Cell-Mediated Cytotoxicity	NA	PMID: 32414908
					PMID: 34339292
Targeting the AGO-KRAS interaction	NA	KRASG12D-driven NSCLC	Ago2 ablation suppresses KRAS signaling in NSCLC	NA	PMID: 33972443
Cyclin-dependent kinase 7 inhibitor	YPN-005	SCLC	YPN-005 significantly decreases the phosphorylation of the carboxyl-terminal domain of RNA polymerase II	NA	PMID: 34224696
Rescuing histone and DNA hypomethylation	SAM	SCLC	SAM rescues KMT2C-loss-initiated epigenetic reprogramming	Clinical trial registration: NCT02535507	PMID: 35449309
pan-HER receptor tyrosine kinase inhibitor	Pyrotinib	HER2 exon 20 insertions in NSCLC	NA	NA	PMID: 30596880
WEE1 Kinase Inhibitor	AZD1775	LKB1-Deficient NSCLC	Loss of ATM phosphorylation of LKB1 contributes to AZD1775 + cisplatin sensitivity	NA	PMID: 28652249
MEK inhibitor	Trametinib, selumetinib	KRAS mutation and amplification NSCLC	NA	NA	PMID: 31694835
Combining FGFR and MEK inhibitors	BGJ398+trametinib combination	FGFR1 amplified LUSC	NA	NA	PMID: 31694835
TMPRSS2 as an attractive pan-coronavirus therapeutic target	NA	pan-coronaviruses	Knockout of TMPRSS2 effectively blocks viral replication	NA	PMID: 34535662
Low-dose IFN pre-treatment	IFN	SARS-CoV-2	NA	NA	PMID: 33128895
Entry inhibitors of SARS-CoV-2	Imatinib, MPA, and QNHC	SARS-CoV-2	Imatinib and QNHC bind with ACE2; MPA and QNHC treatment decrease the expression levels of FURIN	NA	PMID: 33116299
Blocking SARS-CoV-2 infection	GW6471	SARS-CoV-2	GW6471 inhibits the HIF1ɑ-glycolysis axis	NA	PMID: 34731648
Pan-ErbB inhibitor	Lapatinib	SARS-CoV-2	ErbB4 is required for SARS-CoV-2 entry	NA	PMID: 34159337
VimIF assembly inhibitor	Withaferin A	IPF	WFA reduces the invasiveness of lung fibroblasts	NA	PMID: 30944258
IL-11 as a therapeutic target	NA	IPF	IL-11 induces fibrosis in WT organoids, while its deletion prevented fibrosis in HPS4−/− organoids	NA	PMID: 31216486
ALK5 inhibitor and integrin ɑVβ6 antagonist	SB525334, GSK3008348	IPF	ALK5 inhibitors and integrin αVβ6 antagonists suppress TGFβ signaling	NA	PMID: 34798066
Notch2 blockade as a therapeutic strategy	NA	GCM (such as asthma, COPD, and CF)	Antibodies that specifically inhibit Notch2, inhibit IL-13-driven goblet cell metaplasia *in vitro* and *in vivo*	NA	PMID: 25558064

NA, not available; VimIF, vimentin intermediate filament; MPA, mycophenolic acid; QNHC, quinacrine dihydrochloride; SAM, S-Adenosyl methionine; SCLC, small cell lung cancer; NSCLC, non-small cell lung cancer; LUSC, lung squamous cell carcinoma; SARS-CoV-2, severe acute respiratory syndrome coronavirus 2; IPF, idiopathic pulmonary fibrosis; GCM, goblet cell metaplasia; COPD, chronic obstructive pulmonary disease; CF, cystic fibrosis.

#### 7.2.1 Drug testing

During the past decades, scientists have used the traditional 2D culture cell lines to screen antitumor drugs, most of which have proved ineffective in clinical studies (Caponigro and Sellers, 2011). The current research leads us to believe that the organoid technology can fill the gap between classical 2D cell lines and clinical trials. Shi et al. (2020) established a protocol for developing NSCLC organoids to lay the foundation for drug screening and biomarker identification. Jabs et al. (2017) developed an automated microscopy-based assay to resolve drug-induced cell death and proliferation inhibition. This has been used to screen clinically relevant drugs for lung cancer treatment. Kim et al. (2019) created a biobank of 80 lung cancer organoid lines from five subtypes of lung cancer that can be used for anticancer drug screening. Based on genomic alterations, organoids derived from different patients respond differently to drugs: A BRCA2-mutant organoid responds to olaparib, an EGFR-mutant organoid responds to erlotinib, and an EGFR-mutant/MET-amplified organoid responds to crizotinib. Because p.M965I is non-pathogenic to BRCA2 function, organoids with the BRCA2 p.M965I mutation had a higher olaparib IC50 than organoids with the BRCA2 p.W2619C mutation. Using SCLC patient-derived organoids, Choi et al. (2021b). developed a novel CDK7 inhibitor, YPN-005, which showed potent anticancer effects compared to the CDK7 inhibitor THZ1. Human PSC-derived lung organoids infected with SARS-CoV-2 can serve as valuable *in vitro* platforms for drug screening to identify candidate COVID-19 therapeutics. Through high-throughput screening, some FDA-approved drugs, including imatinib, mycophenolic acid, and quinacrine dihydrochloride, have proven to be entry inhibitors of SARS-CoV-2 (Han et al., 2021). Human airway epithelium organoids also were used to test the treatment effect of molnupiravir against different SARS-CoV-2 variants (Lieber et al., 2022). Saul et al. (2021) revealed that Pan-ErbB inhibitors rescued cells from SARS-CoV-2 replication, inflammation and lung injury. Enzalutamide, a second-generation antiandrogen agent, effectively inhibited SARS-CoV-2 infection in human prostate cells. However, enzalutamide showed no antiviral activity in human lung organoids, suggesting its inappropriateness in treating COVID-19 through targeting lung cells (Li et al., 2021b).

In addition to lung cancer and infectious diseases, 3D lung organoids also have been used to develop novel therapeutic strategies for IPF and CF. Using an *in vitro* IPF model, Surolia et al. (2019) observed that vimentin intermediate filament assembly regulated fibroblast invasion in fibrogenic lung injury. Following treatment with withaferin A, an inhibitor of vimentin intermediate filament assembly, 14 of 15 patient pulmospheres demonstrated inhibition of invasion. Chemical screening performed in IPF organoids showed that ALK5 inhibitor and integrin ɑVβ6 antagonist ameliorated the fibrogenic changes (Suezawa et al., 2021). A multimodal iPSC-derived organoid platform was generated for CF drug testing. This platform can be used to accelerate therapeutic development for CF caused by rare variants (Berical et al., 2022). Sodium/glucose cotransporters were revealed as potential therapeutic targets for CF (Hirai et al., 2022).

#### 7.2.2 Personalized medicine

Owing to the heterogeneity of the disease, certain treatments work well for some patients but do not show promising results in others. Personalized medicine applies specific treatments to each patient. PDX model facilitates personalized medicine (Li et al., 2020; Xia et al., 2019; Aboulkheyr Es et al., 2018). However, they have several disadvantages, including a low success rate, high requirements for clinical samples, species differences, and most importantly, the inability to achieve high-throughput drug screening. Organoids derived from patients recapitulate the *in vivo* characteristics of genomic alterations, morphology, transcriptome, and drug response, even after long-term culture (Pasch et al., 2019). Thus, patient-derived organoids (PDOs) have been used as preclinical models to develop personalized medical strategies that provide support for clinical trials. Liello et al. reported a clinical case in which a patient with NSCLC responded significantly to the anti-PD-1 drug pembrolizumab. In concordance with the clinical response, in treating patient-derived 3D spheroids, researchers observed a high sensitivity to immunotherapy, whereas they did not see a significant response to standard cisplatin-based chemotherapy (Di Liello et al., 2019). Similarly, using organoids derived from a HER2-A775_G776YVMA-inserted advanced lung adenocarcinoma patient sample, Wang et al. (2019) demonstrated the antitumor activity of pyrotinib. In phase II clinical trial of pyrotinib 400 mg orally daily, 15 patients with HER2-mutant NSCLC showed an objective response rate of 53.3% and a median progression-free survival of 6.4 months. PDO-based drug testing revealed that the combination of FGFR and MEK inhibitors showed better treatment effects than a single FGFR inhibitor in FGFR1 amplified LUSC (Shi et al., 2020). PDO models from NSCLC patients with EGFR exon 20 insertions were used to characterize the antitumor activity of amivantamab. As observed in the organoid model, amivantamab has potent antitumor activity in NSCLC patients with EGFR exon20ins disease (Yun et al., 2020; Park et al., 2021). In LKB1-Deficient NSCLC organoids, the WEE1 kinase inhibitor AZD1775 alone and in combination with DNA agents has significant anticancer effects (Richer et al., 2017), thus providing a potential clinical application for AZD1775 in LKB1-Deficient NSCLC. Lung adenocarcinoma patient derived organoids were used to identify effective anti-cancer drugs poziotinib and pralsetinib for *ERBB2* exon 20 insertions and *RET* fusions, respectively (Kim et al., 2021). Given the low success rates and the lengthy time in establishing PDOs, Hu et al. (2021) developed an integrated superhydrophobic microwell array chip, which could facilitate high-throughput 3D culture and drug test of lung cancer organoids within a week. For targeted therapy or chemotherapy, the drug test results are in good agreement with clinic outcomes. These findings highlight the important translational nature of organoid-based personalized medicine.

#### 7.2.3 Immunotherapy

Over the past decade, immunotherapy has shown substantial clinical activity for a subset of cancer patients (Riley et al., 2019; O'Donnell et al., 2019). Immune checkpoint inhibitors (ICIs), particularly inhibitors of the PD-1 axis, have led to the increased overall survival of patients with NSCLC (Doroshow et al., 2019; Ko et al., 2018; Gandhi et al., 2018). However, the patient’s response to ICIs depends on the expression of target molecules and the tumor microenvironment. Platforms that allow the analysis and modeling of the complexity of the microenvironment would greatly contribute to the understanding of the critical mechanism that mediates a successful antitumor immune response (Dijkstra et al., 2018; Yuki et al., 2020; Wu et al., 2022). Several recent studies have successfully established *in vitro* culture systems to induce and analyze the tumor immune microenvironment (Bar-Ephraim et al., 2020). Lung organoids have also been used to study NSCLC immunotherapy *in vitro*. Dijkstra et al. (2018) developed a co-culture strategy of autologous tumor organoids and peripheral blood lymphocytes. This method allows tumor-reactive T cells to be enriched from the peripheral blood of patients with NSCLC or CRC. Furthermore, these T cells can kill the matched tumor organoids. This study provides a means to assess the sensitivity and resistance of tumor cells to immunotherapy (Cattaneo et al., 2020). In the same year, Neal et al. (2018) used a patient-derived organoid ALI system to model the tumor immune microenvironment, in which the original tumor T cell receptor spectrum was preserved and the immune checkpoint was blocked with anti-PD-1 and/or anti-PD-L1. Using this method, PDO tumor-infiltrating lymphocytes from NSCLC recapitulated the PD-1-dependent immune checkpoint. Thus, PDOs that incorporate immune and other stromal components may contribute to personalized immunotherapy for cancer. Hai et al. generated genetically engineered mouse lung organoid models of squamous cell lung cancer. Using this genetically defined mouse model and 3D tumor organoid culture system, they revealed that WEE1 inhibition can enhance the antitumor activity of anti-PD-1 monotherapy. Combined immune checkpoint blockade and WEE1 inhibition enhances NK and T cell recruitment in tumors and decreases immunosuppressive neutrophilic tumor infiltration (Hai et al., 2020), suggesting that organoid technology can also be used in the study of combinatorial immunotherapy.

## 8 Limitations and perspectives

Although lung organoids have a wide range of applications, the limitations and challenges of this technology cannot be ignored. The first limitation is the contamination of the lung cancer organoids. Dijkstra et al. (2020) obtained >70 lung cancer organoids derived from patients with NSCLC and identified the tumor purity of organoids by genetic analysis, histomorphology, and immunohistochemistry. They found that eighty percent of organoids derived from intrapulmonary lung cancers were overgrown by normal airway organoids. The establishment rate of pure NSCLC organoids is as low as 17%. Another study reported similar establishment rates (28%) for metastatic NSCLC (Sachs et al., 2019). Contamination has also been described in the culture of prostate cancer organoids (Drost et al., 2016; Gao et al., 2014). The low establishment rate and frequent overgrowth of normal airway organoids limit the potential use of NSCLC organoids for personalized medicine. Strategies to apply growth factor conditions that selectively isolate tumor cells may overcome this complication (Bose et al., 2021). Sachs et al. (2019) used Nutlin-3a, driving TP53 wild-type organoids into senescence or apoptosis, to allow the outgrowth of tumor organoids with mutant TP53 (Vassilev et al., 2004).

Secondly, most current lung organoid models cannot totally mimic native tissue architecture and associated tissue microenvironments (Kim et al., 2020b). In other words, it is difficult to faithfully recapitulate the cell-cell/cell-matrix interactions, which are required to maintain *in situ* organization in tissues (Yin et al., 2016). The current culture conditions are unavailable for self-renew and assembly of some tissue components, particularly nervous cells. One possible solution is to develop and optimize the co-culture systems. Combined with the use of novel materials, the tissue-engineering approach recapitulated *in vivo* tissue development by reconstituting stem cells with other cell components, such as fibroblasts and immune cells. Workman et al. (2017) combined human PSC-derived neural crest cells and human intestinal organoids to recapitulate normal intestinal enteric nervous system development. Similarly, as the frequent impact on the central nervous system by SARS-CoV-2 infection, a 3D neural-perivascular “assembloid” containing pericyte-like cells and cortical organoids were generated. Compared to traditional cortical organoids, assembloids produce approximately 50-fold increased viral particles, showing robust SARS-CoV-2 infection. Assembloids can serve as models for SARS-CoV-2 neural infection and viral neuropathology (Wang et al., 2021). Kim et al. (2020b) created multilayer bladder assembloides by reconstituting the tissue stem cells with stromal components. Normal assembloids recapitulate the *in vivo* architecture with the inner epithelium and connective stroma surrounded by an outer muscle layer. Tumor assemblies represent the pathophysiological features of urothelial carcinoma. In the future, the establishment and optimization of co-culture systems that recapitulate the characteristics of the original tissue will be important for studying lung development and disease.

Despite these limitations, lung organoids have emerged as versatile and powerful *in vitro* models for studying lung diseases. *In vitro* organoids closely recapitulate the genomics and biology of patient tissues and reproduce clinical responses to chemotherapy and immunotherapy, making them highly efficient models for drug development in personalized medicine. Combined with gene-editing technologies, such as CRISPR/Cas9, suppressor genes of organoids can be precisely edited, laying a foundation for research of gene-driven diseases. In summary, overcoming the current limitations and challenges may expand the scope of application and increase the precision of the model.
